# Immunomodulatory effects of atorvastatin on peripheral blood mononuclear cells infected with *Mycobacterium tuberculosis*

**DOI:** 10.3389/fimmu.2025.1597534

**Published:** 2025-07-03

**Authors:** Solima Sabeel, Bongani Motaung, Mumin Ozturk, Trevor S. Mafu, Robert J. Wilkinson, Friedrich Thienemann, Reto Guler

**Affiliations:** ^1^ Department of Pathology, Division of Immunology, Faculty of Health Sciences, Institute of Infectious Diseases and Molecular Medicine (IDM), University of Cape Town, Cape Town, South Africa; ^2^ International Centre for Genetic Engineering and Biotechnology (ICGEB), Cape Town Component, Cape Town, South Africa; ^3^ Division of Experimental Medicine, Department of Medicine, Zuckerberg San Francisco General Hospital, University of California, San Francisco, San Francisco, CA, United States; ^4^ Wellcome Discovery Research Platforms for Infection, Centre for Infectious Diseases Research in Africa, Department of Medicine, Faculty of Health Sciences, Institute of Infectious Disease and Molecular Medicine (IDM), University of Cape Town, Cape Town, South Africa; ^5^ Francis Crick Institute, London, United Kingdom; ^6^ Department of Infectious Diseases, Imperial College London, London, United Kingdom; ^7^ General Medicine & Global Health (GMGH), Department of Medicine and Cape Heart Institute, Faculty of Health Science, University of Cape Town, Cape Town, South Africa; ^8^ Department of Internal Medicine, University Hospital Zurich, University of Zurich, Zurich, Switzerland; ^9^ Cape Universities Body Imaging Centre (CUBIC), University of Cape Town, Cape Town, South Africa

**Keywords:** *Mycobacterium tuberculosis*, atorvastatin, peripheral blood mononuclear cells, apoptosis, mevalonate

## Abstract

**Background:**

Tuberculosis (TB) remains a major global health threat, contributing substantially to high morbidity and mortality rates. This underscores the urgent need for more effective interventions. Recent studies highlight the potential of host-directed therapy approaches to enhance immune defences against TB. Atorvastatin, recognized for both its lipid-lowering properties and its immunomodulatory effects, has emerged as a compelling candidate for host-directed therapy against TB. Here, we investigated the *ex vivo* efficacy of atorvastatin in inducing immunomodulatory activities (phagosome maturation, autophagy, and apoptosis) and enhancing the mycobacterial killing capacity in *Mycobacterium tuberculosis* (*Mtb*)-infected peripheral blood mononuclear cells (PBMCs).

**Method:**

Blood samples from healthy donors were collected for PBMC isolation. PBMCs were then treated overnight with or without atorvastatin, followed by infection with *Mtb* strains (H37Rv, HN878, and CDC1551) to evaluate intracellular mycobacterial growth by colony-forming units enumeration. Furthermore, co-localization of late endosomal marker (Rab-7), lysosomal markers (Cathepsin-D and LAMP-3), and autophagy marker (LC3B) with GFP-*Mtb* was investigated in infected PBMCs using laser scanning confocal microscopy. Moreover, multiple apoptotic assays were performed, including the TUNEL assay for DNA fragmentation, quantification of caspase-3 activity, and the expression levels of the pro-apoptotic gene (*Bax*) and anti-apoptotic gene (*Bcl2*).

**Results:**

Treatment with atorvastatin significantly reduced intracellular mycobacterial replication compared to untreated controls in *Mtb*-infected PBMCs. Moreover, atorvastatin enhanced co-localization between *Mtb* and late endosomal marker (Rab-7), lysosomal markers (Cathepsin-D and LAMP-3), and autophagy marker (LC3B) in *Mtb*-infected PBMCs. Furthermore, atorvastatin robustly promoted apoptosis in *Mtb*-infected PBMCs, as demonstrated by TUNEL assay and caspase-3 activation.

**Conclusion:**

Our findings highlight atorvastatin’s potential as a crucial modulator of the immune response in *Mtb*-infected PBMCs, supporting its role in host-directed therapy.

## 1 Introduction

Tuberculosis (TB) continues to exert a significant toll as the leading cause of mortality among infectious lung diseases worldwide. According to the World Health Organization’s Global Tuberculosis Report 2024, TB claims an estimated 1.3 million lives annually, with 10.8 million new cases reported in 2023 globally (1). In addition, approximately a quarter of the world’s population is inferred to be infected with *Mycobacterium tuberculosis* (*Mtb*), as measured by a positive interferon-gamma release assay or a positive tuberculin skin test. While 5–10% of new infections progress to active disease, this risk increases substantially in immunocompromised individuals, particularly those co-infected with HIV (1, 2).

Treatment of drug-susceptible TB with current drugs requires at least six months to achieve a durable cure, but the prolonged duration significantly increases the risk of non-adherence and treatment failure (3). Low adherence increases the risk of poor outcomes, including treatment failure, relapse, and the development of drug resistance (3). Together with treatment failure and adverse effects, these unintended treatment consequences affect a substantial proportion of patients and have further consequences. For example, adverse treatment effects may lead to severe post-TB lung diseases such as chronic obstructive pulmonary disease, bronchiectasis, and fibrocavitation, among others, which reduce the quality and expectancy of life among treated patients, and increase the likelihood of recurrent TB (4). Advanced therapies, such as targeted therapies, repurposed antimicrobials, and long-acting formulations, are under investigation to improve treatment outcomes (5). Host-directed therapies (HDTs), including immunotherapeutics and non-antimicrobial agents, enhance the host’s immune response against *Mtb* (6, 7). These approaches leverage specific signaling pathways, existing antibiotics, or reduced dosing frequency (6, 7).

Several studies have demonstrated the potential of statins as HDT for TB (8–11). Statins, known as 3-hydroxy-3-methyl glutaryl coenzyme A reductase (HMG-CoA) inhibitors, are primarily prescribed to reduce serum cholesterol levels by inhibiting HMG-CoA reductase. This enzyme drives the conversion of HMG-CoA into mevalonate, a key step in the cholesterol biosynthesis (12). Inhibiting this pathway decreases cholesterol biosynthesis and reduces the synthesis of isoprenoid intermediates such as farnesyl pyrophosphate (FPP) and geranylgeranyl pyrophosphate (GGPP) (13). In the context of *Mtb* infection, statins have shown potential to enhance the bactericidal potency of the first-line anti-TB treatments, and thereby shorten the duration of treatment (14). In preclinical studies, mice treated with simvastatin or rosuvastatin exhibited a reduced *Mtb* burden in the spleens, livers, and lungs when compared to untreated controls (11). Mice treated with simvastatin also showed accelerated clearance of bacilli from the lungs when combined with standard TB therapy (15). Dutta et al. (2020) demonstrated that statins possess potent adjunctive activity against *Mycobacterium tuberculosis*, significantly reducing lung CFU counts in both the standard mouse model (BALB/c mice) and in C3Heb/FeJ mice, a model that more closely resembles human necrotic TB granulomas (16). More recently, Davuluri et al. (2023) reported that atorvastatin enhances the efficacy of first-line anti-TB regimens and markedly reduces the intracellular bacterial burden in the spleens of guinea pigs (8). Additionally, atorvastatin improves the permeability and distribution of rifampicin and isoniazid within granulomatous lung lesions by decreasing granuloma vascular thickness (8).

In clinical studies, peripheral blood mononuclear cells (PBMCs) isolated from individuals with familial hypercholesterolemia treated with atorvastatin exhibited a significant decrease in the intracellular growth of *Mtb* compared to PBMCs from healthy controls (11). In a recent clinical trial, Adewole et al. (2023) found that adjunctive atorvastatin therapy accelerates *M. tuberculosis* clearance in patients with pulmonary TB (17). In contrast, the ROSETTA trial, which evaluated rosuvastatin (10 mg once per day for 8 weeks) as an adjunctive TB therapy, found no significant impact on time to culture conversion and no effect on lung inflammation as measured by PET/CT (18, 19).

Beyond the reported antimicrobial activity, statins also exhibit notable immunomodulatory effects. In a study by Bedi et al. (2017), treatment with 80 mg/day of atorvastatin (considered high-intensive statin therapy) for three months reduced serum levels of interleukin 8 (IL-8), tumor necrosis factor (TNF), intercellular adhesion molecule 1 (ICAM1), C-reactive protein (CRP), and neutrophil counts in patients with bronchiectasis (20). In corroboration, evidence from a pilot study by Mroz et al. (2015) found that atorvastatin 40 mg daily for 12 weeks reduced serum CRP levels, sputum neutrophil count in lung biopsies, and the expression of key genes involved in inflammation and leukocyte activation (21).

Mechanistically, our group previously demonstrated, through the work of Parihar and Guler et al. (2013), that simvastatin promotes phagosome maturation in *Mtb*-infected PBMCs, as indicated by increased EEA-1 and LAMP-3 expression, as well as enhanced autophagy marked by LC3-II accumulation (11). Similarly, Guerra-De-Blas et al. (2019) showed that simvastatin induces apoptosis in *Mtb*-infected PBMCs (22). Additional evidence from Dutta et al. (2016) demonstrated that pravastatin enhances phagosome maturation in *Mtb*-infected mouse bone marrow-derived macrophages (BMDMs) (16). Furthermore, Bruiners et al. (2020) revealed that simvastatin induces autophagy in *Mtb*-infected human PBMCs via blocked activation of mechanistic target of rapamycin complex 1 (mTORC1), activated AMP-activated protein kinase (AMPK) through increased intracellular AMP: ATP ratios, and favored nuclear translocation of transcription factor EB (TFEB) (10). While most mechanistic studies of *Mtb* infection have mainly focused on simvastatin, data on the immunomodulatory effects of atorvastatin—an extensively prescribed statin with a distinct pharmacological profile—remain limited.

Herein, we investigated the immunomodulatory activities of atorvastatin, focusing on its ability to enhance phagosome maturation, autophagy, and apoptosis in *Mtb*-infected PBMCs. Additionally, we assess the mycobacterial killing capacity of atorvastatin-stimulated PBMCs following *ex vivo Mtb* infection.

## 2 Material and methods

### 2.1 Participant samples and ethics

Blood samples were collected from 10 healthy donors (18–65 years, both sexes), according to South African Guidelines for Good Clinical Practice. The study was approved by the University of Cape Town’s Faculty of Health Sciences Human Research Ethics Committee [Reference number: 732/2015].

### 2.2 Isolation of PBMCs

All blood samples were collected in sodium heparin vacutainer tubes (BD Vacutainer^®^, BD Diagnostics, Franklin Lakes, NJ, USA) by a qualified and authorized staff member. The detailed protocol for the isolation of PBMCs is provided in the **Supplementary Material**.

### 2.3 *Ex vivo* infection of PBMCs with *Mtb*

PBMCs were seeded in 96-well plates (Corning^®^, New York, United States) and treated with varying concentrations of atorvastatin (ATO) at 37°C with 5% CO_2_. Following overnight incubation, PBMCs were infected with H37Rv, HN878, and CDC1551 *Mtb* strains with a multiplicity of infection (MOI) of 0.5. Each CFU experiment included three technical replicates per condition. Further details of the experimental protocol can be found in the **Supplementary Material**.

### 2.4 Immunofluorescence staining for confocal microscopy

Atorvastatin-treated and untreated PBMCs were seeded in 96-well plates (ibidi ^®^, Gräfelfing, Germany) with transparent bottoms. Details regarding the confocal microscopy and image-capturing procedures are included in the **Supplementary Material**. The concentration and dilution of primary and secondary antibodies used for immunofluorescent staining were outlined in **Supplementary Table 1**.

### 2.5 DNA fragmentation (TUNEL assay)

*In situ* apoptosis was detected using Click-It™ Plus TUNEL Assay labelled with Alexa Fluor™ 647 picolyl azide dye (Invitrogen, Massachusetts, United States). All the steps were carried out following the manufacturer’s instructions, as explained in the **Supplementary Material**. The mean fluorescent intensity (MFI) was calculated in 3–5 fields that contain 25–30 cells.

### 2.6 Measurement of caspase-3 activity

The proteolytic activity of caspase-3 was determined using the EnzChek^®^ Caspase-3 Assay Kit #2 (Molecular Probes, Leiden, Netherlands), following the manufacturer’s instructions.

### 2.7 Enzyme-linked immunosorbent assay

Supernatants from *ex vivo* cell cultures were collected for evaluation of pro-inflammatory cytokines, including IL-1β, IL-6, and IL-8, through ELISA after atorvastatin stimulation and *Mtb* infection. PBMC were stimulated overnight with different atorvastatin concentrations, including 50 µM, and 100 µM prior to infection with three *Mtb* strains (H37Rv, HN878, and CDC1551). Culture supernatants were collected at 1, 3, and 6 dpi; ELISA plates were read using a VersaMax™ microplate reader (Molecular Devices, USA).

### 2.8 RNA extraction and cDNA synthesis

Atorvastatin-treated and *Mtb*-infected PBMCs were lysed using 2-β-mercaptoethanol (Sigma-Aldrich, St. Louis, Missouri, United States) in lysis buffer (1:100). Total cellular RNA was isolated from cell lysates using the RNeasy Mini kit (Qiagen, Hilden, Germany) according to the manufacturer’s specifications. Total RNA was reverse transcribed using the Transcriptor First Strand cDNA Synthesis Kit (Roche, Basel, Switzerland) according to the manufacturer’s specifications.

### 2.9 Real-time quantitative PCR

Quantitative real-time PCR [LightCycler^®^ 480 II system combined with the LightCycler^®^ 480 SYBR Green I Master (Roche, Manheim, Germany)] was performed to measure the relative expression levels of *Bcl-2* and *Bax* using specific primer pairs (Integrated DNA Technologies, Iowa, United States). Hypoxanthine-guanine phosphoribosyltransferase 1 (HPRT1) was used as the reference housekeeping gene for normalization. Further details can be found in the **Supplementary Material**. All primer sequences and cycling conditions for qPCR are shown in **Supplementary Tables 2** and **3**, respectively.

### 2.10 Statistical analysis

Statistical analysis was performed using GraphPad Prism version 8 (GraphPad Software, San Diego, California, United States). For each experiment, we used two–three biological replicates (different donors) and performed three technical replicates per condition. Student’s *t*-test was used to compare differences in quantitative data between two groups, while one-way ANOVA, with Bartlett’s test for homogeneity of variances, was used for comparisons involving three or more groups. Statistically significant values were represented as *P<0.05, **P<0.01, ***P<0.001 and ****P<0.0001. To account for donor variability, all quantitative data were pooled across donors, and statistical analyses were conducted on biological replicates, using mean values of technical replicates to represent each donor. When applicable, we used one-way ANOVA to determine statistical significance across treatment groups, and all results are presented as mean ± SEM.

## 3 Results

### 3.1 Atorvastatin exhibits a dose-dependent suppression of intracellular *Mtb* growth in *ex vivo Mtb-infected* PBMCs

**Figure 1:**
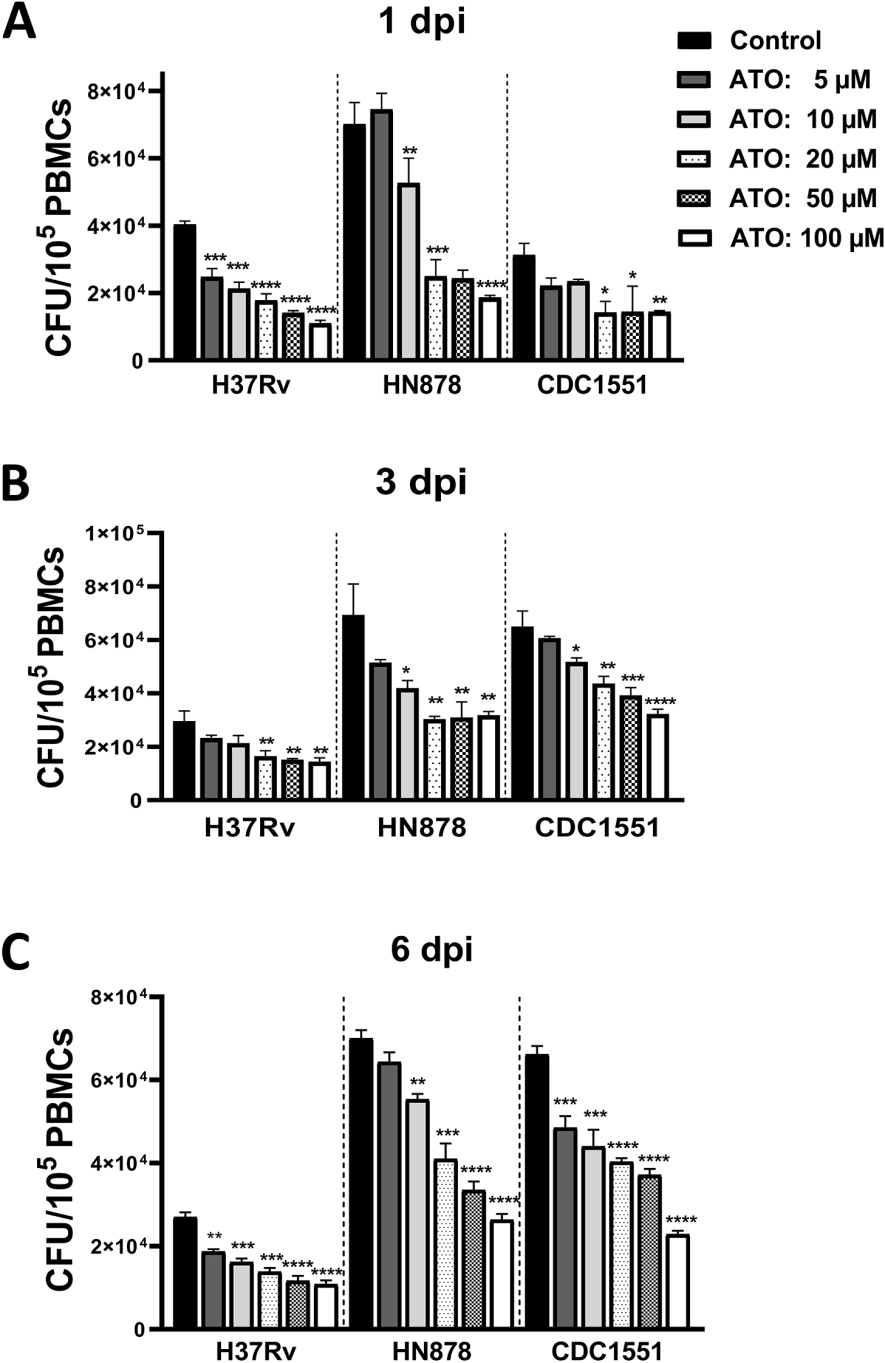
Atorvastatin reduces the intracellular growth of *Mycobacterium tuberculosis* in PBMC. PBMCs were cultured overnight in the presence of atorvastatin (ATO) or control (DMSO). Subsequently, PBMCs were infected with *Mtb* strains (H37Rv, HN878, and CDC1551). **(A)** At 1 day post-infection (dpi), **(B)** 3 dpi, and **(C)** 6 dpi, PBMCs were lysed to measure the intracellular growth of *Mtb* using CFU assay. All data are shown as mean ± SEM using one-way ANOVA with Bartlett’s Test. The results were representative of three independent experiments, *P<0.05, **P<0.01, ***P<0.001, and ****P<0.0001 compared to the control. ATO, atorvastatin; dpi, days post-infection.

We examined the mycobactericidal killing capacity of atorvastatin in *Mtb*-infected PBMCs. We demonstrated that, compared with untreated controls, atorvastatin significantly reduced the intracellular growth of *Mtb* in a dose-dependent manner, at 1, 3, and 6 days post-infection (**Figures 1A-C**).

### 3.2 Atorvastatin enhances phagosome maturation and autophagy in *Mtb*-infected PBMCs

**Figure 2:**
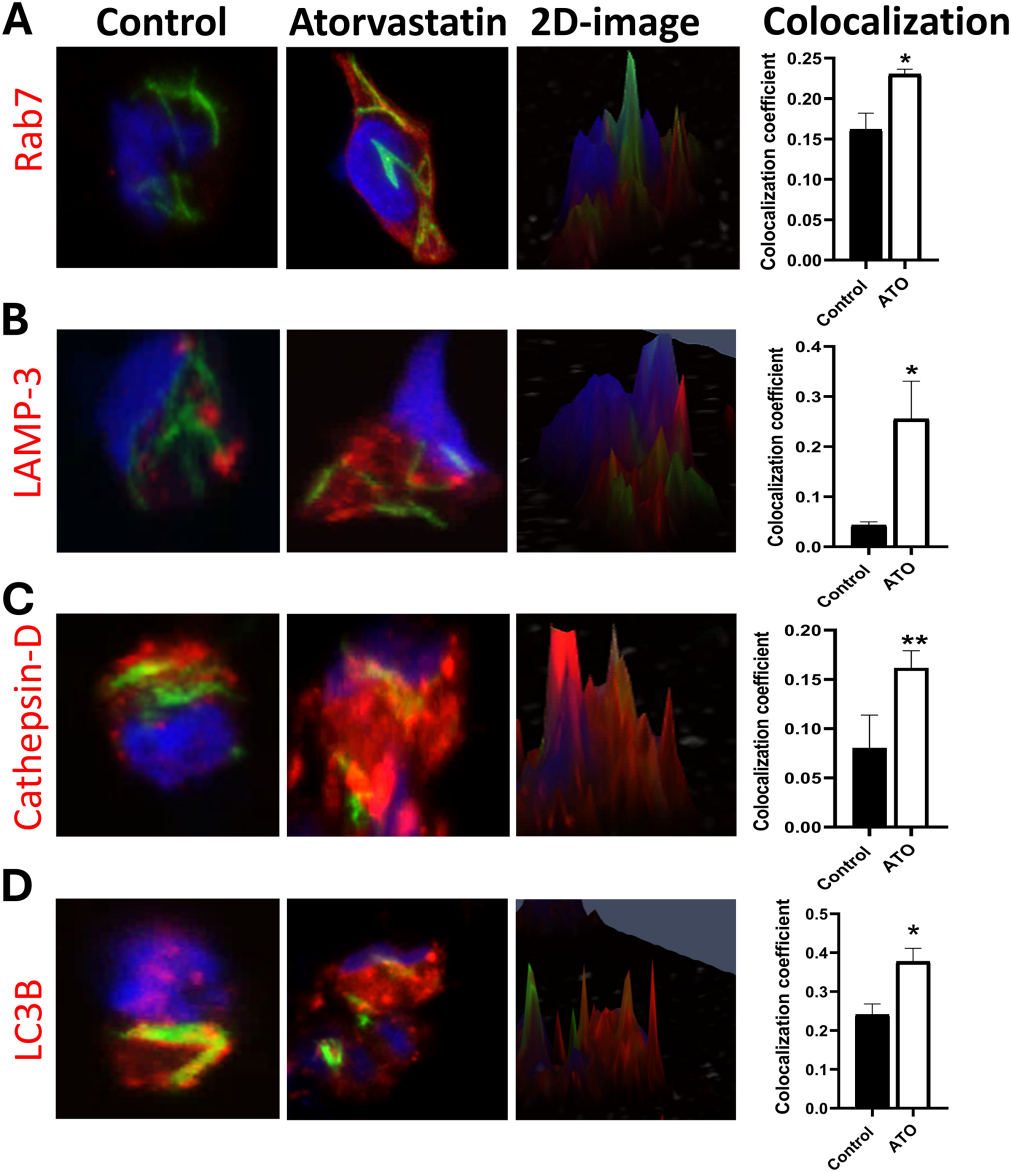
Atorvastatin promotes phagosome maturation and autophagy in PBMCs by increasing colocalization of late endosomal, lysosomal, and autophagy markers with *Mtb.* Confocal microscopy images and two-dimensional images of atorvastatin-treated PBMC showing enhanced co-localization of GFP-expressing *Mtb* H37Rv (green) with **(A)** late endosomes (Rab-7), **(B, C)** phagolysosome markers (LAMP-3, Cat-D), and **(D)** autophagy marker LC3B. Quantitative analysis using Spearman’s Rank co-localization coefficient showing increased colocalization in atorvastatin-treated PBMCs following *Mtb* infection. The yellow color represents the colocalization of green (GFP-*Mtb*) with red (phagosome and autophagy markers), and blue indicates nuclear stain (Hoechst stain). Data are represented as mean ± SEM (*P<0.05, **P<0.01). ATO, atorvastatin.

To evaluate the impact of atorvastatin on these processes, we measured key markers of phagosome (Rab-7), phagolysosome (LAMP-3 and Cathepsin-D), and autophagy (LC3B) maturation in GFP-H37Rv *Mtb*-infected PBMCs using confocal microscopy and quantitative image analysis. Atorvastatin treatment significantly increased the colocalization of *Mtb* with the late endosomal marker (Rab-7), phagolysosome markers (LAMP-3 and Cathepsin-D), and the autophagy marker (LC3B) in GFP-H37Rv *Mtb*-infected PBMCs (**Figures 2A-D**). Further image quantification using Spearman’s Rank co-localization coefficient confirmed atorvastatin’s ability to increase the colocalization of *Mtb* with these key phagosome and autophagy markers. Collectively, these findings suggest that atorvastatin is associated with enhanced host protective functions, including increased phagosome maturation and augmented autophagy, which may contribute to the restriction of intracellular *Mtb* growth.

### 3.3 Atorvastatin enhances apoptosis in *Mtb*-infected PBMCs by inducing *in situ* DNA fragmentation and activating caspase-3

**Figure 3:**
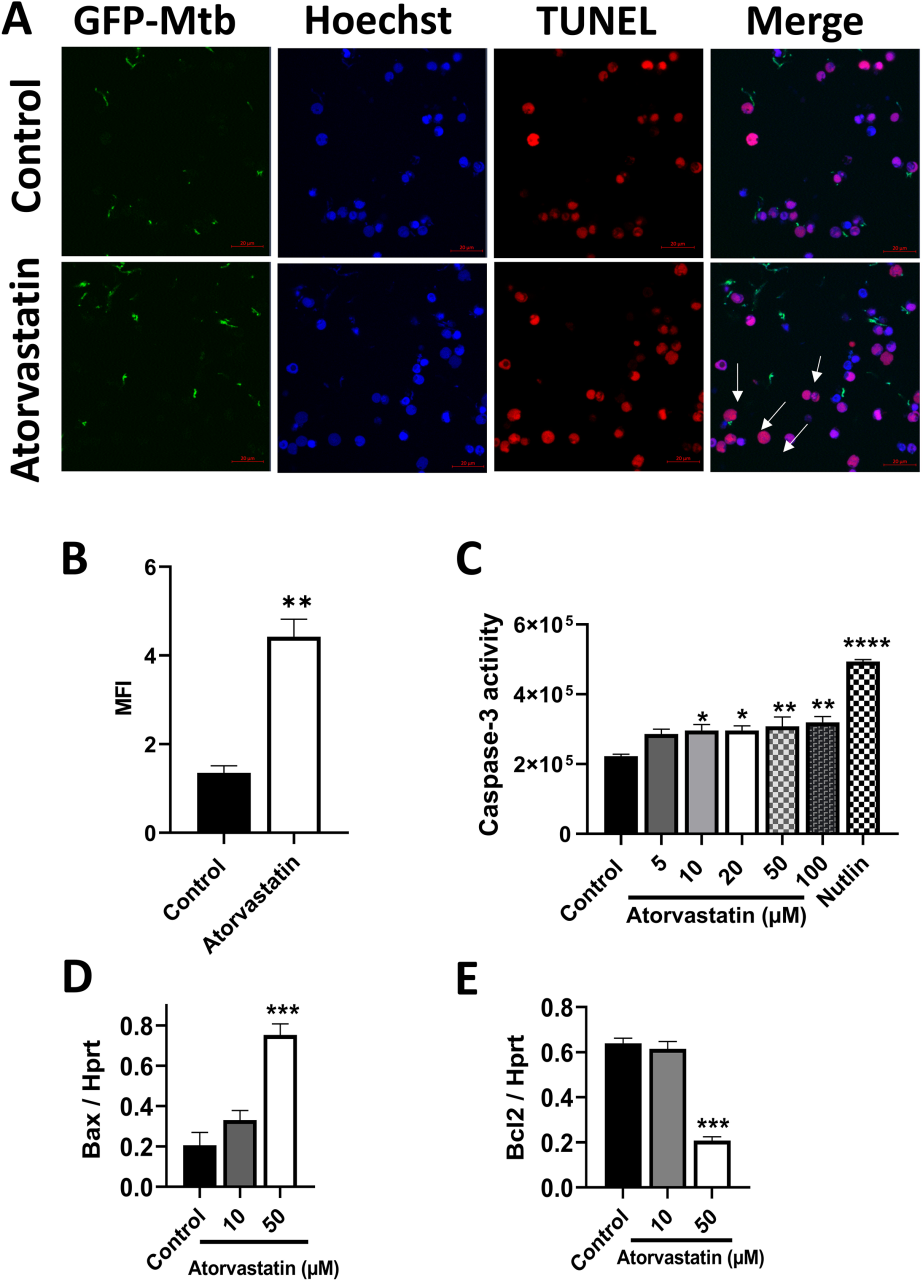
Atorvastatin enhances apoptosis in *Mtb*-infected PBMCs as measured by TUNEL and caspase-3 activity. PBMCs (5x10^5^/well) were treated with atorvastatin and DMSO (control) overnight, followed by infection with GFP-expressing H37Rv *Mtb* at an MOI:5 (1 PBMC: 5 *Mtb*) for 24 hours to assess apoptosis by TUNEL assay. **(A)** Confocal microscopy images depicting GFP-expressing H37Rv *Mtb* in green, Hoechst nuclear stain in blue, and TUNEL-positive apoptotic cells in red. White arrows indicate apoptotic cells, which appear magenta in the overlay of nuclear Hoechst stain (blue) and TUNEL-positive cells (red). The scale bar represents 20 μm. Images were acquired using an Airyscan 880 confocal microscopy with a 63x oil immersion lens. **(B)** Quantitative analysis of the mean fluorescence intensity (MFI) of Tunnel-positive PBMCs. **(C)** PBMCs (2x10^6^/well) were treated with various concentrations of atorvastatin (5 μM, 10 μM, 20 μM, 50 μM, and 100 μM), DMSO (control), and the apoptosis inducer Nutlin overnight to assess caspase-3 activity using the Ac-DEVD-CHO caspase assay kit, presented as fluorescence unit. PBMC were treated with atorvastatin and infected with *Mtb* H37Rv for 24 hours to measure **(D)** the pro-apoptotic marker *Bax* and **(E)** the anti-apoptotic marker Bcl-2 using RT-qPCR. Data are presented as mean ± SEM (*P<0.05, **P<0.01, ***P<0.001, and ****P<0.0001).

Macrophages play a crucial role in innate defense against *Mtb* by inducing apoptosis (23). To investigate atorvastatin’s ability to enhance apoptosis as a host defense mechanism to control *Mtb* proliferation, we performed three assays on PBMCs: a TUNEL assay, a caspase-3 activity assay, and an expression assay evaluating the levels of pro- and anti-apoptotic markers. Atorvastatin treatment induced pro-apoptotic characteristics, as measured across all three assays. TUNEL-positive cells were significantly increased in atorvastatin-treated PBMCs compared with untreated controls, as evidenced by the overlay of the TUNEL-positive signal (magenta) with the Hoechst-counterstained nuclei (blue) and TUNEL-positive cells (red, **Figure 3A**). Quantitative image analysis confirmed a threefold increase in apoptotic cells induced by atorvastatin (**Figure 3B**). Caspase-3 activity was significantly increased in atorvastatin-treated PBMCs compared with untreated controls (**Figure 3C**). The pro-apoptotic marker (*Bax*) was significantly upregulated in atorvastatin-treated PBMCs compared with controls (**Figure 3D**), while the anti-apoptotic marker *Bcl-2* was significantly downregulated following atorvastatin treatment (**Figure 3E**). Taken together, these data suggest that atorvastatin induces apoptosis as a host protective mechanism in *Mtb*-infected PBMCs.

### 3.4 Mevalonate supplementation reverses the effect of atorvastatin and increases the intracellular growth of *Mtb*

**Figure 4:**
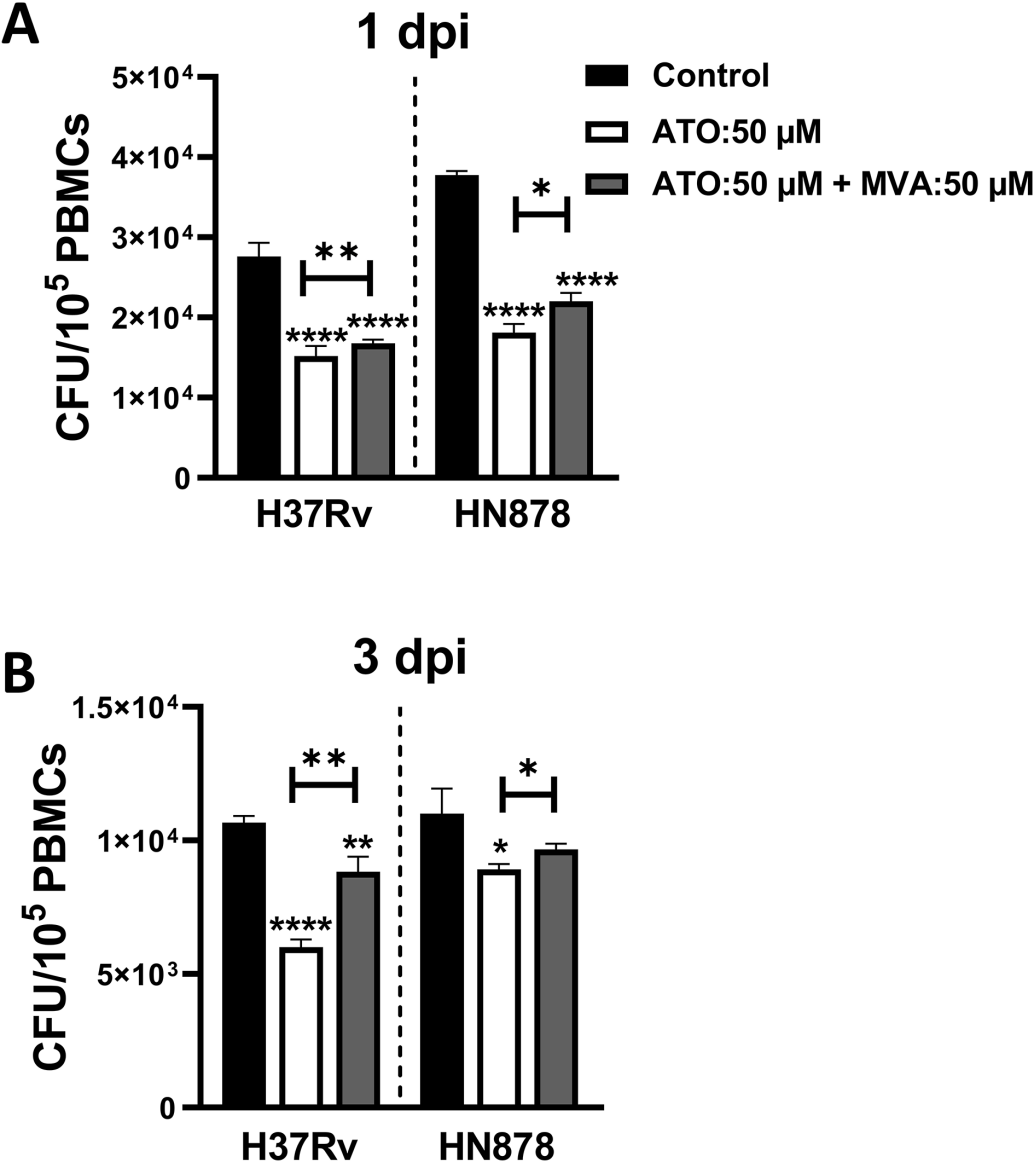
Mevalonate supplementation reverses the effect of atorvastatin and enhances the intracellular growth of *Mtb* in PBMCs. PBMCs (1x10^5^ cells/well) were treated with either atorvastatin (50 μM), DMSO control, or a combination of atorvastatin (ATO, 50 μM) and mevalonate (MVA, 50 μM). Following overnight treatment, PBMCs were infected with *Mtb* H37Rv or HN878 strain for **(A)** 1 dpi and **(B)** 3 dpi to evaluate the intracellular *Mtb* growth using CFU assays. Data shown are presented as mean ± SEM (*P<0.05, **P<0.01, ****P<0.0001). ATO, atorvastatin; MVA, mevalonate; dpi, days post-infection.

To investigate atorvastatin’s ability to directly reduce intracellular growth of *Mtb* through inhibiting the cholesterol biosynthesis pathway, we supplemented *Mtb*-infected PBMCs with exogenous mevalonate, a precursor downstream of HMG-CoA reductase in the cholesterol biosynthesis pathway. The addition of mevalonate partially reversed the atorvastatin-induced reduction in CFU, with a notable effect observed in the H37Rv strain at 3 dpi (**Figures 4A, B**).

### 3.5 Atorvastatin reduces intracellular *Mtb* growth mainly through inhibition of the geranylgeranyl biosynthesis

**Figure 5:**
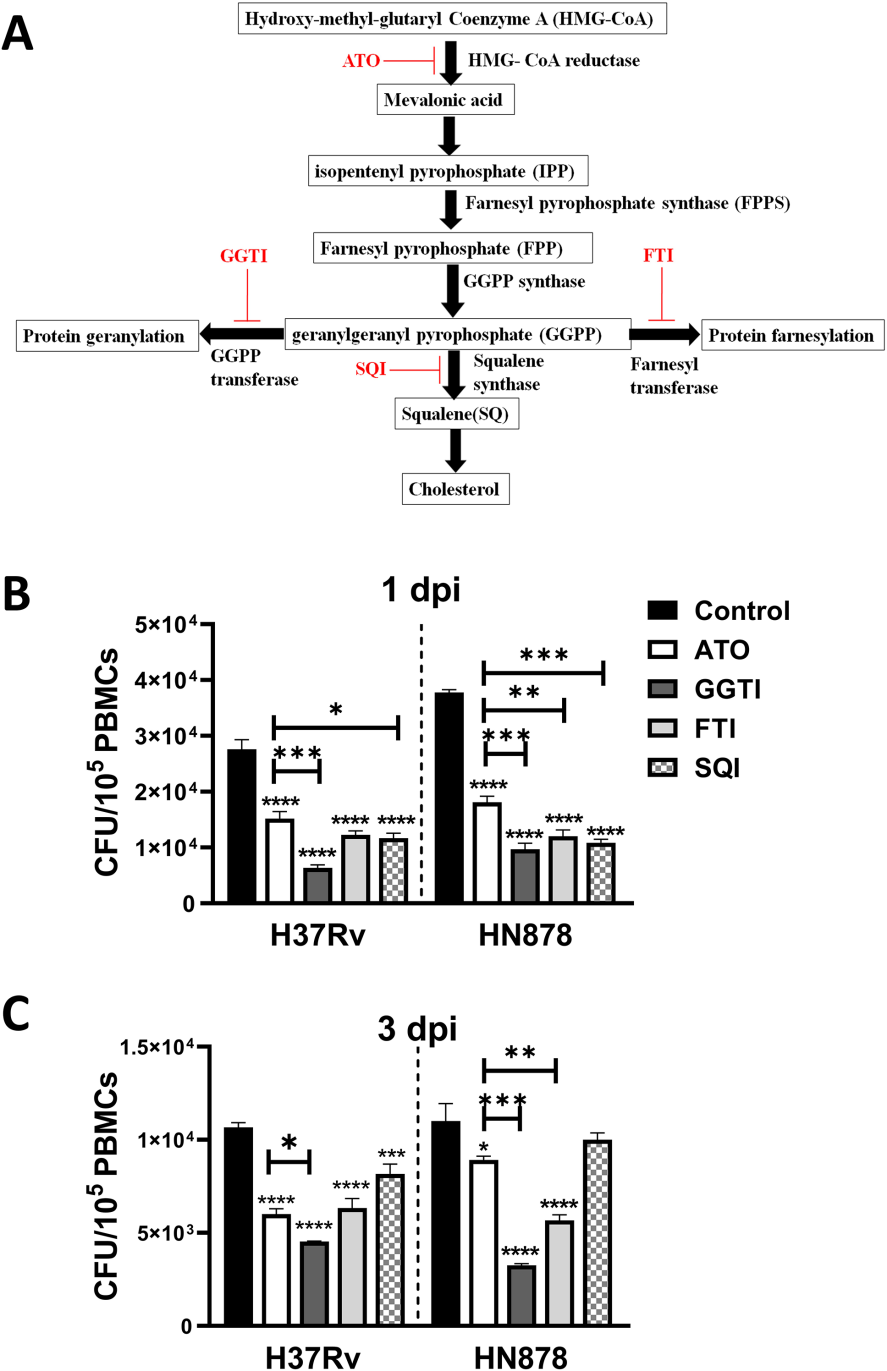
Inhibition of the protein geranylation pathway significantly reduces intracellular growth of *Mtb* in PBMCs. Schematic representation of the mevalonate pathway highlighting key metabolites, enzymes, and specific inhibitors (ATO, atorvastatin; FTI, farnesyl transferase inhibitors; GGTI, geranylgeranyl transferase inhibitors; SQI, squalene inhibitors). **(A)** PBMCs were treated overnight with atorvastatin (50 μM) or specific mevalonate pathway inhibitors (GGTI, FTI, and SQI, 50 μM each). Treated PMCs were then infected with *Mtb* H37Rv or *Mtb* HN878 strain for **(B)** 1 dpi and **(C)** 3 dpi to measure the intracellular *Mtb* growth using CFU assay. Data are presented as mean ± SEM (*P<0.05, **P<0.01, ***P<0.001, and ****P<0.0001). dpi, days post-infection.

The mevalonate pathway is a vital biochemical process responsible for synthesizing cholesterol and metabolites for protein geranylation and farnesylation, critical processes involved in signal transduction and membrane anchoring (24). Atorvastatin inhibits HMG-CoA reductase, the enzyme that catalyzes the conversion of HMG-CoA to mevalonate, thereby reducing cholesterol synthesis. Consequently, atorvastatin also blocks metabolites for protein geranylation and protein farnesylation. To investigate the precise metabolic axis within the mevalonate pathway that contributes to the atorvastatin-mediated reduction of intracellular *Mtb* growth, we used specific inhibitors of the mevalonate pathway. PBMCs were treated with geranylgeranyl transferase inhibitor (GGTI), farnesyltransferase inhibitor (FTI), or squalene inhibitor (SQI), and then infected with either H37Rv or HN878 *Mtb* strains to evaluate the intracellular mycobacterial growth (**Figure 5A**). Early during *Mtb* infection, at 1-day post-infection, all three inhibitors (GGTI, FTI, and SQI) significantly reduced the intracellular growth of *Mtb*, even surpassing the efficacy of atorvastatin treatment (**Figure 5B**). In contrast, at 3 days post-infection, the geranylgeranyl transferase inhibitor exhibited the most substantial reduction in intracellular *Mtb* growth in PBMCs compared to the other inhibitors (**Figure 5C**). In summary, atorvastatin’s host-mediated reduction of intracellular *Mtb* growth is attributed not only to cholesterol inhibition but also to its ability to reduce metabolites in the protein geranylation and protein farnesylation pathway, particularly within the geranylgeranyl biosynthesis pathway.

### 3.6 Atorvastatin reduces IL-1β secretion in a concentration- and time-dependent manner

Atorvastatin treatment reduced IL-1β secretion in a concentration- and time-dependent manner in *Mtb*-infected human PBMCs, regardless of the infecting strain. In contrast, IL-6 and IL-8 levels remained largely unchanged (**Supplementary Figure 5**).

## 4 Discussion

In this study, we investigated the effect of atorvastatin on the *ex-vivo* intracellular growth of *Mtb* in PBMCs. Our findings revealed that pre-treatment of *Mtb*-infected PBMCs with atorvastatin reduces the intracellular growth of *Mtb* - across three strains: H37Rv, HN878, and CDC1551 - in a dose-dependent manner, compared with untreated controls (**Figures 1A-C**). This observation is consistent with a previous study conducted by Lobato et al. (2014), which demonstrated that both cholesterol-lowering drugs: atorvastatin and simvastatin, reduce the intracellular growth of *Mtb* H37Rv in THP-1 derived macrophages (25). Additionally, similar outcomes were reported by Parihar and Guler et al. (2013), who observed a decrease in *Mtb* H37Rv growth in simvastatin-treated murine macrophages (11). Further supporting of these findings, *ex vivo* treatment of PBMCs derived from TB patients with persistent lung inflammation (defined as Total Lung Glycolysis [TLG] ≥ 50 SUV*mL) at the end of TB treatment significantly reduced the intracellular growth of *Mtb*, as measured by CFU assay at 3 dpi. This reinforces the potential of atorvastatin as a host-directed therapy, particularly in TB patients with residual inflammatory activity post-treatment **Supplementary Figure 1**. At Day 0 (4 hours post infection), all three *Mtb* strains showed equal bacterial load **Supplementary Figure 2**.

Macrophages can limit the intracellular growth of *Mtb* through various mechanisms including phagosome acidification and fusion of phagosomes with lysosomes (26). Phagosome maturation is a complex process involving a series of events within the endocytic pathway, transforming a newly formed phagosome into a phagolysosome capable of degrading engulfed microbes and other particles (27). During phagocytosis, the small GTPase Rab-5 recruits early endosomal antigen-1 (EEA-1) to the membrane of phagosomes (28, 29), then another GTPase, Rab-7, is recruited in the later stages of the endosomal trafficking (30, 31). As phagosomes mature, phagolysosomes occur through fusion with lysosomal vesicles that acquire lysosomal markers, such as lysosome-associated membrane protein (LAMP), and acid hydrolyses, such as cathepsin D (Cat-D) (26, 32). Considering Rab-7’s essential role in endosomal trafficking and lysosome biogenesis (33), we hypothesized that atorvastatin might promote the recruitment of phagosome/phagolysosome maturation markers such as Rab-7, LAMP-3 and Cat-D to *Mtb*-contained endosomes. We observed a notable enhancement in the co-localization of Rab-7, LAMP-3, and LC3B with *Mtb* following atorvastatin treatment (**Figure 2A**). Similarly, we observed increased co-localization of LAMP-3 and Cat-D with GFP-*Mtb* in atorvastatin-treated PBMCs compared with untreated controls (**Figures 2B, C**). Our findings are consistent with a previous study by our group, Parihar and Guler et al. (2013), which reported higher co-localization of EEA-1, LAMP-1, and LAMP-3 with GFP-H37Rv *Mtb* in simvastatin-treated murine macrophages (11). Considerable evidence highlights the critical role of autophagy in the host defense against *Mtb* infection (34, 35). Autophagy facilitates the delivery of cytosolic macromolecules and organelles containing *Mtb* to lysosomes for degradation (36). In this context, the role of LC3B, a reliable autophagy marker (37), was assessed. Our data demonstrated an increase in the colocalization coefficient of GFP-*Mtb* with LC3B in atorvastatin-treated cells when compared to controls (**Figure 2D**). Together, these results confirm that atorvastatin enhances the host-mediated killing effector mechanisms of PBMCs, thereby reducing the mycobacterial burden. By enhancing phagosome maturation and autophagy, atorvastatin may counteract the inhibition or impairment of these processes by *Mtb* as an immune evasion strategy (38). To improve transparency and interpretability, the corresponding single-channel images have been included as **Supplementary Figure 3**.

Apoptosis plays a fundamental role in host defense against *Mtb* by reducing the viability of intracellular pathogens (23, 39, 40) and preventing the release and spread of *Mtb* (41). Therefore, we investigated whether atorvastatin induces apoptosis in *Mtb*-infected PBMCs. One hallmark of apoptosis is chromatin fragmentation and alterations in nuclear organization, which can be visualized using the TUNEL assay (42). **Figures 3A, B** demonstrate that atorvastatin induces apoptosis in *Mtb*-infected PBMCs through chromatin fragmentation. This finding is consistent with the observations of Carton et al. (2004), who reported an increase in TUNEL-positive cells following lovastatin treatment in RAW264.7 murine macrophages infected with *Salmonella enterica* serovar Typhimurium (43). These findings illustrate that atorvastatin induces programmed cell death in *Mtb*-infected PBMCs, as a crucial host protective immune response against *Mtb*. Our investigation extended to critical cell signaling events during apoptosis, including caspase-3 activity and regulation of the expression of pro- and anti-apoptosis genes. As shown in **Figure 3C**, atorvastatin increased caspase-3 activity in *Mtb*-infected PBMCs. This observation aligns with findings by Guerra et al. (2019), who noted increased caspase-3 expression in PBMCs treated with simvastatin compared to untreated PBMCs (22). Notably, the pro-apoptotic marker, *Bax*, was significantly overexpressed while the anti-apoptotic marker, *Bcl-2*, was significantly underexpressed in atorvastatin-treated PBMCs compared with untreated controls (**Figures 3D, E**). Our findings mirror those of Blanco-Colio et al. (2002), who reported a reduction in *Bcl-2* expression in vascular smooth muscle cells cultured with atorvastatin and simvastatin in a time- and dose-dependent manner (44). By enhancing apoptosis via inducing DNA fragmentation and activating caspases, atorvastatin offers a potential strategy to subvert *Mtb*’s impairment of the apoptotic immune response (45).

To investigate whether the addition of mevalonate reversed the impact of atorvastatin, a metabolic rescue experiment was conducted. As shown in **Figures 4A, B**, the addition of mevalonate reversed the effects of atorvastatin and enhanced the growth of both H37Rv and HN878 *Mtb* strains. We next investigated the effects of mevalonate pathway inhibitors on Mtb bacterial load. As shown in **Figures 5B, C**, the inhibition of geranylgeranyl pyrophosphate by geranylgeranyl transferase inhibitors significantly contributed to reducing the intracellular growth of *Mtb* in PBMCs. In interpreting our findings on the use of mevalonate pathway inhibitors (**Figure 5**), it is important to consider their potential off-target effects in PBMCs. Inhibitors such as GGTI and FTI disrupt the post-translational prenylation of small GTPases (e.g., Ras, Rho, Rab), which are critical for immune cell signaling, intracellular trafficking, and apoptosis regulation (46). While such inhibition may contribute to the observed bacterial control effects, it may also unintentionally alter broader PBMC functions, including cytokine production (47) and cell proliferation (48). Moreover, the potential for cross-prenylation—where proteins such as Rho undergo geranylgeranylation as a compensatory response to farnesyltransferase inhibition—may confound the interpretation of FTI-specific effect (49). Additionally, SQI, which targets downstream cholesterol biosynthesis, may impair membrane integrity and disrupt receptor-mediated signaling in PBMCs, potentially influencing immune responses independently of *M*. *tuberculosis* infection (50). In summary, our study demonstrates the impact of atorvastatin on enhancing endosomal and phagosome maturation, as well as apoptotic effectors within *Mtb*-infected PBMCs. These findings contrast with those of Bruiners et al. (2020), who reported that inhibition of protein prenylation did not reduce intracellular *Mtb* burden (10). Notably, our study extends these earlier observations by utilizing PBMCs, a more heterogeneous immune cell population compared to the macrophage monolayers used previously. Furthermore, we assessed earlier post-infection timepoints (days 1 and 3), in contrast to the 6-day timepoint examined by Bruiners et al. (2020), providing additional insight into the temporal dynamics of atorvastatin’s immunomodulatory effects (10).

Statins are known to modulate inflammatory responses, partly through inhibition of NF-κB activation, with atorvastatin exhibiting one of the strongest effects among the statin class (51). In the current study, we investigated the impact of atorvastatin on cytokine responses in *Mtb*-infected human PBMCs. Our data show that atorvastatin reduces IL-1β secretion in a concentration- and time-dependent manner. regardless of the infecting *Mtb* strains. This observation is consistent with findings by Ascer et al. (2004), who reported reduced plasma IL-1 levels in hypercholesterolemic patients following atorvastatin treatment (52). In contrast, the levels of IL-6 and IL-8 remained unaffected under the same conditions, as shown in **Supplementary Figure 5**. These data suggest that atorvastatin selectively modulates IL-1β responses, indicating a potentially targeted anti-inflammatory mechanism relevant to host–pathogen interactions in tuberculosis.

In summary, our study demonstrates the impact of atorvastatin on enhancing endosome and phagosome maturation, as well as apoptotic effectors within *Mtb*-infected PBMCs. This combination of mechanisms makes atorvastatin a compelling candidate for host-directed therapy against tuberculosis—the multiple mechanisms exert various effects against *Mtb*, with the potential to act synergistically and reach dormant reservoirs. When used in combination with standard TB therapy, atorvastatin may reduce the risk of relapse, post-TB lung disease, and associated pulmonary vascular and cardiovascular complications, thereby promoting better lung function and long-term health outcomes.

Nonetheless, our study was not without limitations. While we primarily used PBMCs from healthy donors, further investigation is warranted to explore the immunomodulatory effects of atorvastatin therapy in TB patients, thereby providing more robust evidence for its potential as a host-directed therapy for tuberculosis.

## Data Availability

The original contributions presented in the study are included in the article/**Supplementary Material**. Further inquiries can be directed to the corresponding authors.

## Supplementary material

Supplementary Figure 1
*Ex vivo* treatment with atorvastatin of PBMC derived from patients with persistent lung inflammation (TLG ≥ 50 SUV*mL) at the end of TB treatment, significantly reduced the intracellular growth of Mtb strains, as measured by CFU assay at 3 days post-infection.

Supplementary Figure 2 Effect of atorvastatin treatment on intracellular *Mtb* burden at 4 hours post-infection (hpi). PBMCs were infected with *Mtb* strains H37Rv, HN878, or CDC1551 and treated with 50 µM and 100 µM of atorvastatin. CFUs were quantified at 4 hours post-infection (4 hpi) to assess bacterial uptake.

Supplementary Figure 3 Representative single-channel immunofluorescence images. Cells were stained with antibodies against LAMP-3, Rab7, Cathepsin-D (shown in red: Cy3 or Alexa Fluor 594), and nuclei were counterstained with DAPI (blue). GFP-*Mtb* is shown in green. Overlay images display merged channels to visualize co-localization.

Supplementary Figure 4 Screenshot from FUJI software illustrating the optimization parameters used for the Spearman’s rank correlation colocalization analysis.

Supplementary Figure 5 Pro-inflammatory cytokine secretion in *Mtb*-infected PBMCs following atorvastatin stimulation. Cytokines were measured at 1, 3, and 6 dpi using ELISA; **(A)** IL-1β, **(B)** IL-6, and **(C)** IL-8, where the dotted line indicates detection limits. Data is shown as a representative of three replicates and shown as mean ± SD. Data was analysed using Shapiro-Wilk test for normality, followed by two-way ANOVA with Dunnett’s *post hoc* test for multiple comparisons against the control; *p ≤ 0.05, **p ≤ 0.01, ***p ≤ 0.001, ****p ≤ 0.0001. ATO, atorvastatin; DPI, Days Post-Infection; LOD, Limit of Detection.
