# Supplementary material for: Immunomodulatory effects of atorvastatin on peripheral blood mononuclear cells infected with Mycobacterium tuberculosis
Source: Front Immunol. 2025 Jul 3;16:1597534. doi: 10.3389/fimmu.2025.1597534 (PMC12267233; doi:10.3389/fimmu.2025.1597534)
Supplement: Supplementary Figure 1 — Ex vivo treatment with atorvastatin of PBMC derived from patients with persistent lung inflammation (TLG ≥ 50 SUV*mL) at the end of TB treatment, significantly reduced the intracellular growth of Mtb strains, as measured by CFU assay at 3 days post-infection. [file DataSheet1.pdf]

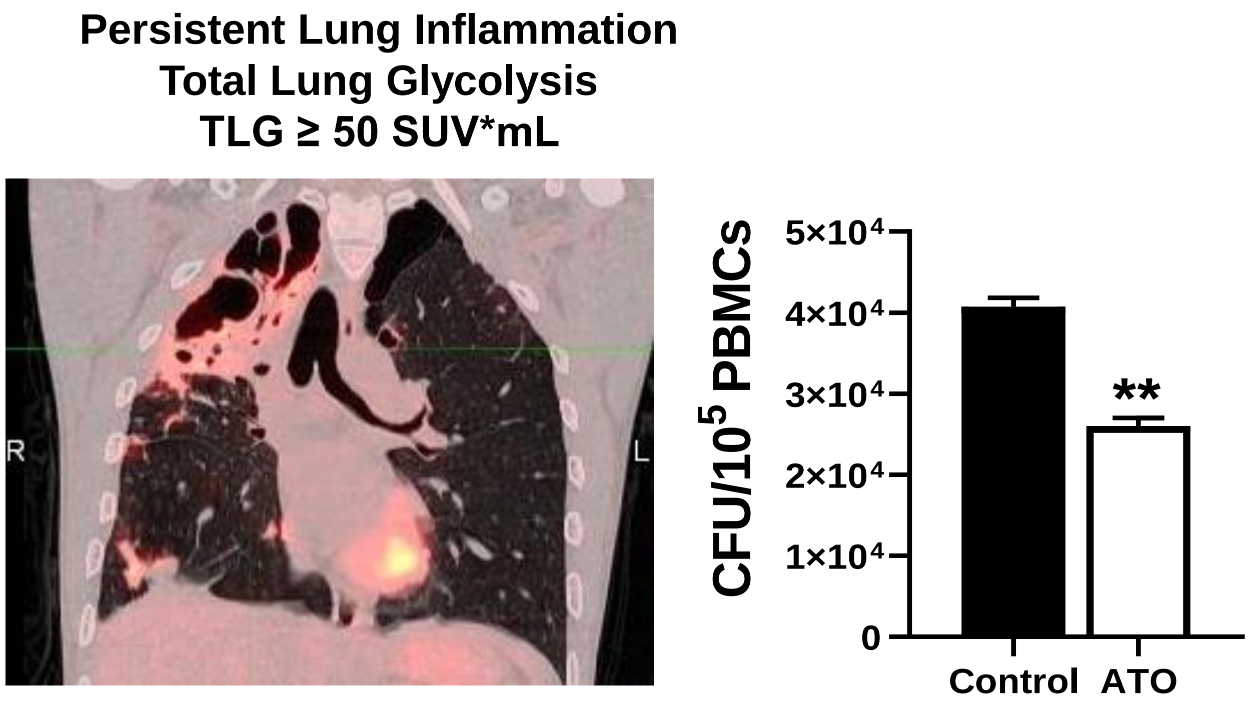

Supplementary Figure 1

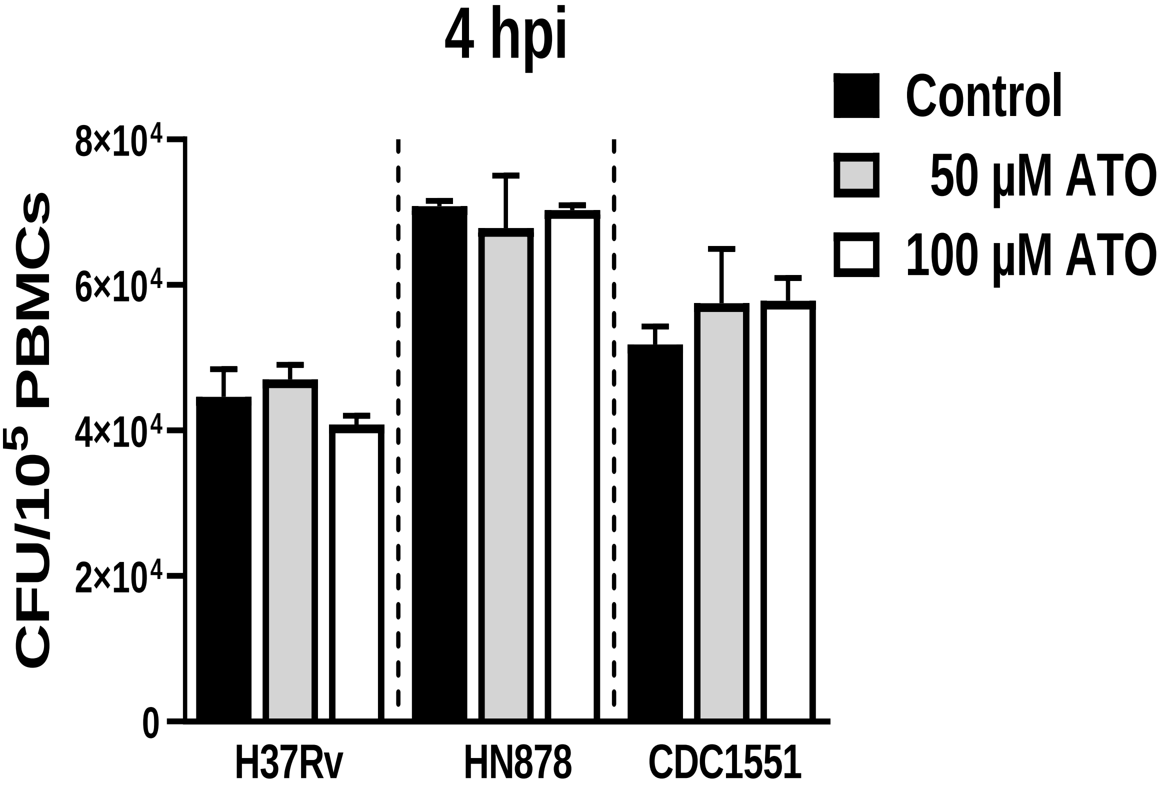

Supplementary Figure 2

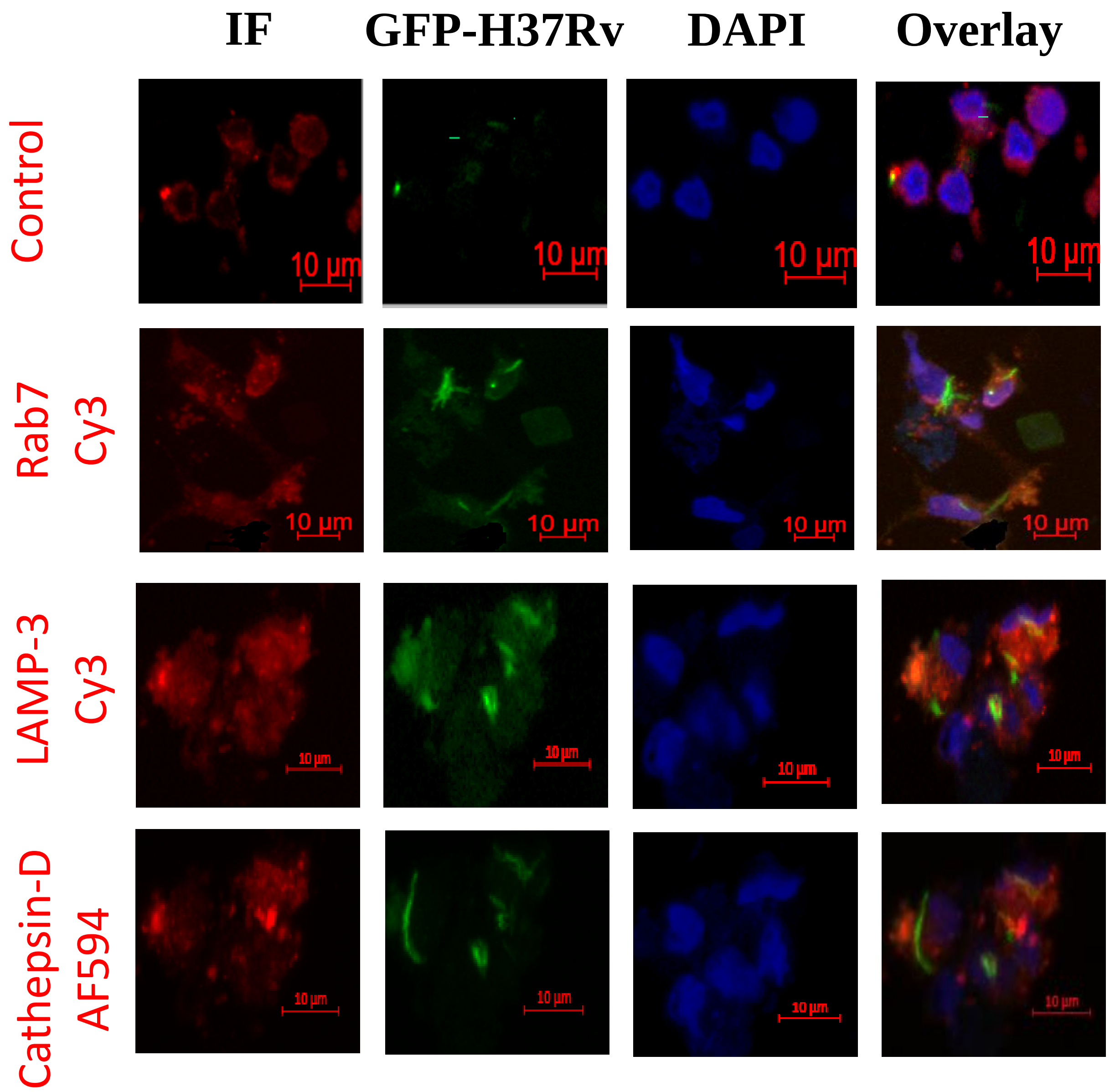

Supplementary Figure 3

Threshold regression: Costes

Pearson's R value (no threshold): 0.65
Pearson's R value (below threshold): 0.60
Pearson's R value (above threshold): -0.51
Spearman's rank correlation value: 0.19464548
Spearman's correlation t-statistic: 101.6014
t-statistic degrees of freedom: 262142.000
Manders' M1 (Above zero intensity of Ch2): 1.000
Manders' M2 (Above zero intensity of Ch1): 1.000
Manders' tM1 (Above auto threshold of Ch2): 0.032
Manders' tM2 (Above auto threshold of Ch1): 0.024

Supplementary Figure 4

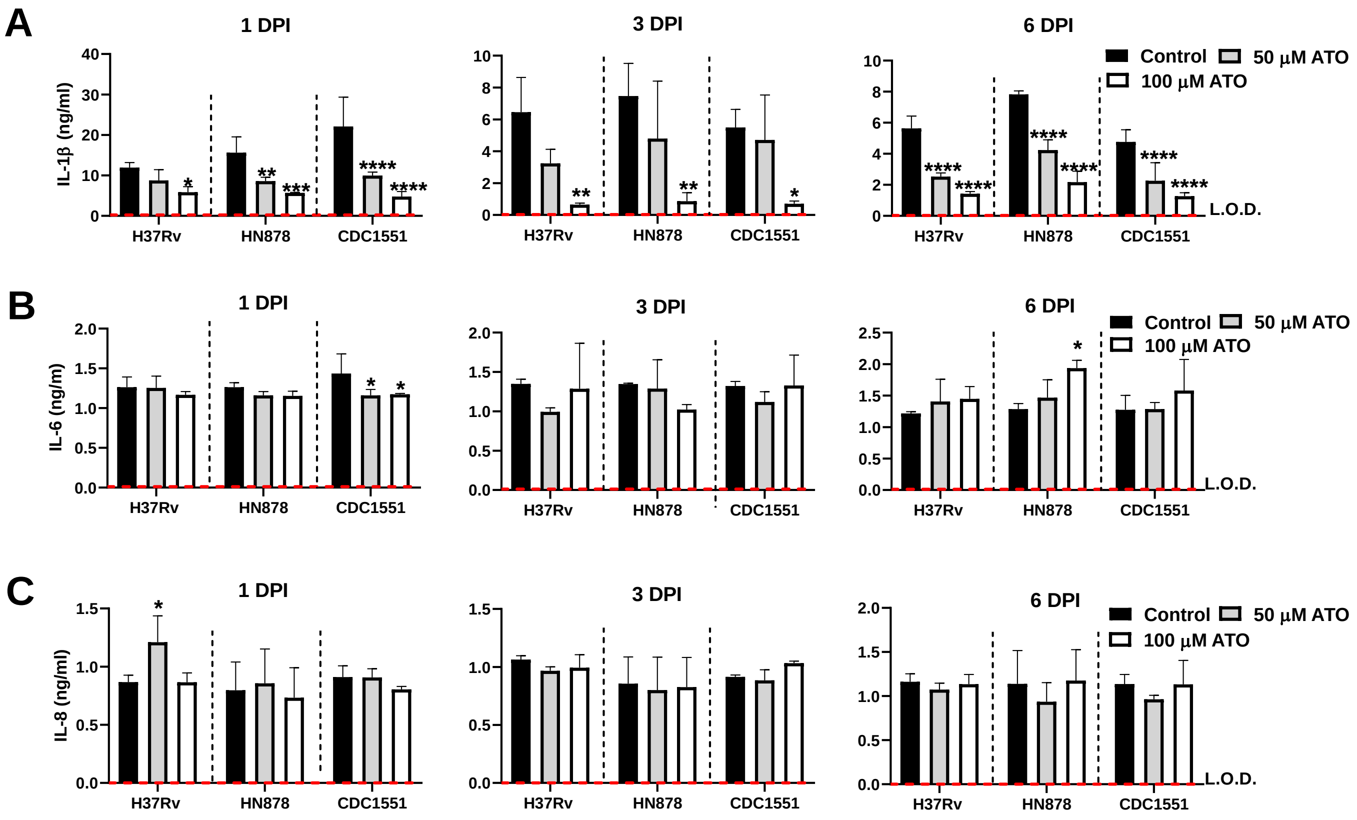

Supplementary Figure 5
