# Supplementary material for: Immunomodulatory effects of atorvastatin on peripheral blood mononuclear cells infected with Mycobacterium tuberculosis
Source: Front Immunol. 2025 Jul 3;16:1597534. doi: 10.3389/fimmu.2025.1597534 (PMC12267233; doi:10.3389/fimmu.2025.1597534)
Supplement: Supplementary file 2 [file DataSheet2.pdf]

Supplementary Tables

Supplementary Table 1: Concentrations of antibodies used for immunofluorescent staining.

Markers	Primary Antibodies	Concentration	Secondary Antibodies	Concentration	Fluorochrome Conjugates
Rab-7	Mouse anti-Rab-7	0.5 µg/ml	Rabbit anti-mouse	1/400	Cy3
Cat-D	Rabbit anti-Cat-D	1/100	-	-	AF594
LAMP-3	Mouse anti-LAMP-3	5 µg/ml	Rabbit anti-mouse	1/400	Cy3
LC3B	Rabbit anti-LC3B	2.5 µg/ml	Goat anti-rabbit	1/500	AF594
Nuclear statin	DAPI<br>Hoechst	1/1000<br>1/5000			

Supplementary Table 2: The nucleotide sequences of primers used for RT-qPCR.

Gene Name	Primer Sequence (5´ - 3´)
hs-Hprt1	Forward: AGG CGA ACC TCT CGG CTT T
	Reverse: AAG ACG TTC AGT CCT GTC CAT
hs-Bcl-2	Forward: TAC AGG CTG GCT CAG GAC TAT
	Reverse: CGC AAC ATT TTG TAG CAC TCT G
Bax-1	Forward: GCT CAA GGC CCT GTG CAC TAA A
	Reverse: TCT TGG ATC CAG ACA AGC AGC CG

Supplementary Table 3: Thermal profile of reverse transcription amplification.

Steps	Cycle	Temperature	Time	Analysis mode
1. Pre-incubation	1	95°C	5 minutes	None
2. Amplification:				
(I) Denaturation	45	95°C	10 seconds	Quantification
(II)Annealing		63°C	15 seconds	
(III)Extension		72°C	15 seconds	
(IV) Acquisition		75°C	1 second	
3. Melting curve analysis:				
(I) Denaturation	1	95°C	5 minutes	Melting curve
(II) Re-Annealing		65°C	1 hour	
(III)Melting		97°C	1 second	
4. Cooling	1	40°C	30 minutes	None
