# Supplementary material for: Immunomodulatory effects of atorvastatin on peripheral blood mononuclear cells infected with Mycobacterium tuberculosis
Source: Front Immunol. 2025 Jul 3;16:1597534. doi: 10.3389/fimmu.2025.1597534 (PMC12267233; doi:10.3389/fimmu.2025.1597534)
Supplement: Supplementary file 3 [file DataSheet3.pdf]

Experimental protocols

Preparation of atorvastatin solution

To prepare the stock solution, 181.9 mg of atorvastatin calcium (Sigma-Aldrich) was dissolved in 750 µl DMSO. A day before usage, the stock solution of atorvastatin (200 mM) was further diluted to appropriate concentrations with RPMI-1640 medium and kept rotating at room temperature overnight.

Preparation of mevalonate

500 mg of mevalonolactone (Sigma-Aldrich) was dissolved in 5 mL of ethanol to make a 778.4 mM stock solution, which was then diluted further to the desired concentration.

Preparation of mevalonate pathway inhibitors

Twenty-five mg of geranylgeranyl transferase inhibitor obtained from Sigma-Aldrich was diluted in 1 ml DMSO to make a 43.8 mM stock solution, then the appropriate concentration was prepared. One mg of farnesyltransferase inhibitor obtained from TOCRIS Bioscience was dissolved in 3.56 ml of water to make a 0.5 mM stock solution, then the appropriate concentration was prepared. 50 mg of squalene inhibitor obtained from Cayman; was dissolved in 2 ml DMSO/PBS (500 DMSO: 1500 PBS) to prepare a 67 mM stock solution. All prepared solutions were filtered before use.

Isolation of peripheral blood mononuclear cells

Under sterile conditions, collected blood was transferred to 50 ml Falcon tubes and diluted 1:1 with RPMI-1640 medium with glutamine (Gibco™, Invitrogen Corporation, Carlsbad, CA, United States). Diluted blood was loaded into 15 ml Ficoll® Paque Plus (Cytiva, Life Sciences, Marlborough, United States) in 50 ml Falcon tubes. Using an Eppendorf® 5810R benchtop centrifuge, the tubes were centrifuged at 1000 x g for 20 minutes (acceleration max and brakes off). The PBMC layer was extracted by Pasteur pipette into a 50 ml Falcon tube, which was

then filled up to 50 ml with RPMI-1640 medium and centrifuged at 120 xg (acceleration max and brakes off) for 10 minutes. In the second washing phase, PBMCs were topped up with 40 ml RPMI-1640 medium and then centrifuged at 120 xg (acceleration max and brakes off) for 10 minutes. PBMCs were resuspended in 1 ml RPMI-1640 medium.

Ex vivo infection of PBMCs with Mtb

Infected PBMCs were incubated at 37°C in an incubator containing 5% CO_2 for the desired time points (1 dpi and 3 dpi). For each time point, infected cells were centrifuged at 500 xg for 5 minutes at 4°C. The pellets were then resuspended in 200 µl of 1x PBS to wash the cells before being centrifuged at 500 xg for 5 minutes at 4°C and discarded supernatant. The cells were then lysed by adding 100 µl of 0.1% Triton X-100. Cell lysate was serially diluted and plated in Middlebrook 7H10 agar enriched with 10% OADC medium and 5% glycerol, and cultured plates were incubated at 37°C under 5% CO2. The growth of *Mtb* strains was estimated through the calculation of the number of CFU for each condition after 14 days.

In vitro infection of PBMCs with green fluorescent protein H37Rv Mtb for confocal microscopy

All infecting procedures were conducted in BSL III. One vial of GFP-H37Rv *Mtb* strain (bacterial stock: 2.4 x 10^7 CFU/ ml) at logarithmic phase was thawed at room temperature, then centrifuged at 10000 rpm for 5 minutes. The supernatant was decanted, and the bacterial pellet was resuspended in 1 ml sterile 1X PBS. Another washing step was performed by centrifugation of the bacterial suspension at 10000 rpm for 5 minutes. Then the pellet was resuspended in 1 ml sterile 1X PBS and transferred to a new Eppendorf tube containing 3 sterile 3 mm glass beads; thereafter, the suspension was vigorously vortexed for 1 min. The suspension was allowed to settle for 30 minutes, then centrifuged at 800 xg for 3 min. The supernatant was sonicated six times (3 seconds plus and 7 seconds rest) using an ultrasonic bath. A multiplicity of infection (MOI:5) was calculated according to the number of seeding cells.

Immunofluorescence staining for confocal microscopy:

The PBMCs were infected with GFP-H37Rv *Mtb*. At 1 dpi, infected cells were fixed by adding 80% cold methanol for 5 minutes at -20°C, then were washed three times with 1X PBS at room temperature for 10 minutes each. Fixed cells were permeabilized using 0.1% Triton X-100 for 5 minutes at room temperature. A blocking buffer (1% BSA / 10% normal goat serum / 0.3 M glycine in 0.1% PBS-Tween) was added to reduce the non-specific binding of antibodies for 1 hour, then cells were washed three times with 1X PBS for 10 minutes each. The cells were then incubated overnight at 4°C with anti-Rab7, anti-LAMP3, anti-Cathepsin D and anti-LC3B primary antibodies (Abcam, Cambridge, United Kingdom) in a sealed humidified chamber. Thereafter, cells were washed three times with 1X PBS at room temperature for 10 minutes each. Secondary antibodies: Cy3^{TM} rabbit anti-mouse IgG (Jackson ImmunoResearch Laboratories, Pennsylvania, United States) and Alexa Fluor™ 546 goat anti-rabbit IgG (Invitrogen, Massachusetts, United States) were added for 90 minutes, then cells were washed three times with 1X PBS for 10 minutes. Counterstain with nuclear stain (DAPI or Hoechst) was added for 10 minutes, then cells were washed once with 1X PBS for 10 minutes at room temperature. Mowiol was added and incubated overnight at room temperature, then the plate was stored at 4°C till viewing by Zeiss laser scanning confocal microscopy (880 Airy Scan).

DNA fragmentation (TUNEL Assay)

Following the manufacturer's instructions, a plate containing atorvastatin-treated and Mtb-infected PBMCs was centrifuged to remove media, and then washed once with 1x PBS. A sufficient volume of the permeabilization reagent (0.25% Triton™ X-100 in PBS) was added, incubated for 20 minutes at room temperature, and then washed twice with deionized water. 100 µl of TdT reaction buffer was added to each well and incubated for 10 minutes at 37°C. For each reaction, 50 µl of TdT reaction mixture (1 µl EdUTP nucleotide mixture, 2 µl TdT enzyme, and 47 µl TdT reaction buffer) was added and incubated for 60 minutes at 37°C in a dark, humidified chamber. Thereafter, the plate was blocked with blocking solution (3% BSA in 0.1% Triton™ X-100 in PBS) for 5 minutes. Following blocking, Click-It™ Plus TUNEL reaction cocktail (10% Click-It™ Plus TUNEL Reaction buffer additive and 90% Click-It™ Plus TUNEL supermix) was added for 30 minutes at 37°C in a dark chamber. Twice washing steps with 3% BSA in 1x PBS for 5 minutes each were conducted to remove Click-It™ Plus TUNEL reaction cocktail. Hoechst nuclear stain was added for 15 minutes at room temperature.

Measurement of the proteolytic activity of caspase-3

Briefly, PBMCs were collected and washed with 1x PBS and lysed using the lysis buffer (200 mM TRIS, Ph 7.5, 2 M NaCl, 20 mM EDTA, 0.2% TRITON™ X-100) for 30 minutes on ice. The lysed cells were centrifuged at 5000 rpm for 5 minutes at 4°C to pellet the cellular debris, and then the supernatant from each sample was collected. A volume of 50 µl aliquots of the extracts were incubated with Z-DEVD Rhodamine 110 substrate (Molecular Probes, Leiden, Netherlands), a specific substrate for caspase 3 at final concentration of 50 µM in 1 ml of reaction buffer [50 Mm PIPES, Ph 7.4, 10 mM EDTA, 0.5% 3-[(3-cholamido-propyl) dimethyl lammonio]-1-propane sulfonate (CHAPS), 1 M dithiothreitol (DTT)] for 30 minutes at room temperature. The amount of Z-DEVD–R110 released was measured on a fluorescence CLARIOstar® Plus Microplate Reader (BMG LABTECH, Württemberg, Germany) at excitation and emission wavelengths of 496 and 520 nm, respectively. The fluorescence was expressed in arbitrary units (AU). The specificity of the reaction was assessed by adding the specific inhibitor of 1 mM Ac-DEVD-CHO inhibitor (Molecular Probes, Leiden, Netherlands).

RNA Extraction

Briefly, an equal volume of 70% ethanol was added to the cell lysate and then spun down by centrifugation at 8000 xg for 20 seconds at 4°C. To ensure that the total RNA was free of DNA, efficient DNase digestion was performed by adding DNase I mixture (10 µl DNase I + 80 µl RDD buffer) for 15 minutes at room temperature. The RW1 buffer was added, spun down at 8000 x g for 20 seconds at 4°C, and then the flow-through was discarded. The previous step was repeated, and subsequent wash steps were carried out using RPE buffer to clean the RNA. Afterwards, the spin column was placed into a new collection tube, RNase-free water was added to the spin column, and then spun down at 8000 x g for 1 minute to elute the RNA. Concentration and purity of extracted RNA were measured at 260/230 and 260/280 wavelengths, respectively, using the ND-1000 NanoDrop spectrophotometer (Thermo Fisher Scientific, Massachusetts, United States).

Quantitative Real-Time PCR Analysis of Gene Expression

The cDNA was added to the PCR mixture (1X Master Mix, 0.25 µM Forward primer, 0.25 µM Reverse primer). Amplification was carried out in the LightCycler® 480 II system combined

with the LightCycler® 480 SYBR Green I Master (Roche, Manheim, Germany) following the manufacturer's guidelines. In addition, levels of hypoxanthine-guanine phosphoribosyltransferase 1 (HPRT1) were used as a reference for normalization. The relative quantification of the transcripts was determined by using the ΔΔCt method; untreated samples were set as the control sample equal to 1 (calibrator).

Image capture and analysis

Stained cells were observed with a Zeiss laser scanning confocal microscope 880 equipped with a plan-apochromat 10x/0.3 objective and a plan-apochromat 63X / 1.4 numerical aperture (NA) oil immersion objective. Samples were excited using 5 laser lines: 405, 458, 488, 514, 561, and 633nm with a Xenon 120 UV lamp. Images were acquired sequentially to avoid bleed-through signals, with a scanning mode format of 480 x 480 pixels, with the highest image quality reaching 140nm resolution. The transmission and detector gains were set to achieve the best signal-to-noise ratios, and the laser powers were tuned to limit the bleaching of fluorescence. The refractive index of the immersion oil used was 1.51 (ZEISS, Württemberg, Germany). All settings were rigorously maintained for all experiments. All images and Z-Stack of three-dimensional data were captured and recorded using ZEN Blue version 3.3 (ZEISS, Württemberg, Germany). All the images were in the TIFF RGB format and Differential Interference Contrast (DIC). The co-localization coefficient of *Mtb* and various biomarkers, and the mean fluorescent intensity were calculated using ZEN blue version 3.3 (ZEISS, Württemberg, Germany) and ImageJ (National Institutes of Health, Maryland, USA), respectively.

Cell viability assay

In a 96-well U-bottom plate, treated cells were centrifuged at 500 xg for 5 minutes at room temperature, and then the cell supernatant was discarded. The cells were resuspended in a mixture of 80 µl of RPMI-1640 medium and 20 µl of CellTiter-Blue® reagent. Cell suspensions were incubated at 37°C for 4 hours in an incubator supplemented with 5% CO_2. The assay plate was shaken for 10 seconds, and then the fluorescence was measured at 560 nm with a reference wavelength of 590 nm using the CLARIOstar® Plus Microplate Reader (BMG LABTECH, Württemberg, Germany) supported by MARS for data analysis and export interfaces.
